# Assessment of the capacity to modulate brain signals in a home-based SMR neurofeedback training setting

**DOI:** 10.3389/fnhum.2022.1032222

**Published:** 2023-01-05

**Authors:** Miriam Autenrieth, Silvia Erika Kober, Guilherme Wood

**Affiliations:** ^1^Institute of Psychology, University of Graz, Graz, Austria; ^2^BioTechMed-Graz, Graz, Austria

**Keywords:** neurofeedback, home-based, mental strategies, EEG, generalization, transfer

## Abstract

Electroencephalogram (EEG)-based neurofeedback (NF) is mainly used in clinical settings as a therapeutic intervention or to optimize performance in healthy individuals. Home-based NF systems are available and might facilitate general access to NF training, especially when repeated training sessions are necessary. However, it remains an open question whether NF training at home is possible without remote monitoring. In the present study, we assessed the capacity of healthy individuals to modulate their own EEG activity when using a home-based NF training system in a comparable manner as if participants had purchased a commercially available NF system. Participants’ face-to-face contact with experimenters was reduced to a minimum, and instructions were provided only in the form of written information or videos. Initially, 38 participants performed 9 sessions of sensorimotor rhythm (SMR) (12–15 Hz) based NF training (three generalization sessions, six training sessions). An active control group (*n* = 19) received feedback on random EEG frequencies. Because of technical problems, bad EEG data quality, or non-compliance, 21 participants had to be excluded from the final data analysis, providing first evidence for the difficulties of non-supervised home-based NF training. In this study, participants were not able to modulate their own brain activity in a desired direction during NF training. Our results indicate that personal interaction with a NF expert might be of relevance and that remote supervision of the training data and more direct communication with the NF users are necessary to enable successful NF training performance. We provide suggestions for the development and implementation of home-based NF systems.

## 1. Introduction

Neurofeedback (NF) allows individuals to gain control over their brain activity. Therefore, a person’s brain activity [e.g., with the help of the electroencephalogram (EEG)] is recorded, analyzed, and presented in real-time to individuals to learn how to modulate their brain activity (Coben and Evans, 2010). Modulating brain activity can positively impact cognitive functions and behavior (Kropotov, 2009; Gruzelier, 2014a). EEG NF is used as a therapeutic intervention (Omejc et al., 2019) and to optimize performance in healthy individuals (Gruzelier, 2014a).

Generally, NF is time and cost-intensive. For instance, up to 50 sessions might be necessary to improve ADHD symptoms (Arns et al., 2009), and two to five training sessions per week for six to 18 months might be necessary to reduce epileptic seizure rates (Tan et al., 2009). Attaching EEG electrodes and operating the feedback system is generally done by an NF trainer, therapist, or experimenter (Strehl, 2020). Therefore, NF users have to come multiple times to a clinic or an EEG laboratory to perform the training. Clearly, these circumstances limit potential NF users’ access to NF training.

In this context, tele-rehabilitation (TR) offers a less time and cost-consuming opportunity for NF training. TR is the transfer of necessary rehabilitation or training interventions from the clinical environment to the patient’s home environment. Outsourcing these interventions improves access to healthcare for people who live in rural areas or have limited physical mobility (Brennan et al., 2009). Clinical outcomes of TR evaluation studies are promising: TR interventions are comparable with face-to-face interventions regarding physical, functional, and psychological measures. Additionally, patients and therapists seem satisfied with these interventions (Kairy et al., 2009). There is even evidence that TR interventions are less expensive than traditional rehabilitation interventions (Grona et al., 2018).

In this regard, it is essential to distinguish between NF as TR intervention and home-based NF systems. TR interventions are continuously monitored by therapists/trainer, NF users get feedback about EEG data quality and an online interaction with the NF therapist/trainer, e.g., *via* video chat function, is possible (Kober et al., 2016, 2019). Some TR interventions are offered as continuing home treatment after in-person sessions. Patients are trained in the use of the TR system by experts/therapists during their in-person sessions to ensure correct use of the TR at home. In contrast, commercially available home-based NF systems are available either with online therapist monitoring or without monitoring. All types of applications can potentially extend the accessibility, usability, and affordability of NF-based interventions: it requires less mobility, is reachable for individuals with a lower degree of functionality in daily living, and the flexibility in the training schedule allows daily training sessions, if required, and thereby increases the compliance to rather extended training programs (e.g., with 40, 80, or even 120 sessions).

Nevertheless, home-based NF applications, especially un-monitored systems, may also hide some drawbacks. One challenge is the correct montage of the sensors, and another is the sensitivity to artifacts, which may be much lower in consumer-grade, unsupervised home-based EEG systems compared to medical-grade EEG systems used in clinics or laboratories by EEG experts (Ratti et al., 2017). Also, the training environment cannot be controlled as comprehensively as in a professional setting. For example, electric devices (computers, TVs, or even power sockets) can cause artifacts at a specific frequency (e.g., 50 Hz), or individuals may be distracted by the surroundings in the room (other people, running TV or radio, stuffed desk). Finally, the effectiveness of home-based training may diminish without guided instructions and continuously motivating participants, especially when training reaches stagnation.

Although the number of commercially available neurofeedback training (NFT) systems that allow home-based training is increasing, there are only a few empirical studies evaluating the usability, efficacy, and training outcome of home-based NFT. For example, Klicken oder tippen Sie hier, um Text einzugeben.Klicken oder tippen Sie hier, um Text einzugeben. showed that TR-NFT is able to decrease sleep latency and improve sleep quality in patients with Insomnia. In another study, Kober et al. (2019) demonstrated that TR-NFT has a positive impact on cognitive functions in patients with Multiple Sclerosis. In both the Cortoos et al. (2010) and Kober et al. (2019) studies, NFT sessions were continuously monitored remotely by NF experts. Two studies evaluated the training outcome of commercially available home-based NF systems: Krepel et al. (2022) used a home-based NF system, where all instructions during the home-based NFT were provided by a mobile NF system. Participants trained four times per week on their own at home, every fifth session was done in the clinic supervised by a NF expert. The home-based NFT led to improvements in sleep quality (Krepel et al., 2022). Birch et al. (2022) trained their participants remotely *via* a video link before the beginning of the NFT until they were able to mount the EEG headset, minimize EEG artifacts, and complete a full training session on their own. The home-based NFT sessions were performed without assistance from a supervising NF expert. Home-based NFT resulted in a significant pain reduction in patients with chronic pain (Birch et al., 2022). The results of these studies provide evidence for the successful regulation of one’s own brain activity through home-based NFT.

Interestingly, in former home-based NFT studies, the percentage of NF non-learners seems to be higher compared to laboratory studies. Kober et al. (2019) reported that half of their participants did not learn to modulate their brain activity and Krepel et al. (2022) stated that 21 of 37 participants were classified as non-learners. In comparison, the number of non-learners is often much smaller in laboratory studies (Zoefel et al., 2011; Enriquez-Geppert et al., 2014) and more in line with the general assumption that about 15–30% of NF users are not able to modulate their brain activity (Allison and Neuper, 2010). Additionally, in all the above-cited home-based NFT studies, NF experts monitored, trained, or supervised participants. At this point, we are unaware of any study that assessed individuals’ capacity to modulate their brain signals through home-based NFT without the assistance of a therapist or NF expert. Nevertheless, commercially available NF systems are advertised as non-medical products that offer the possibility of modulating everyone’s brain signals and improving everyone’s daily life functions (e.g., sleep or concentration) without the necessity of an expert, what is a problematic claim (Neubauer and Wood, 2022).

The aim of the present study was to assess the capacity of healthy individuals to modulate their own brain signals without expert guidance in a home-based NFT setting. Therefore, participants performed a sensorimotor rhythm (SMR)-based NFT on their own at home, in a similar way as if they had purchased an unsupervised home-based NF system, over nine sessions. We chose an SMR-based NFT because increasing the SMR (12–15 Hz) is one of the most frequently used NFT protocols to improve cognitive function in healthy individuals and clinical populations (Kropotov, 2009; Gruzelier, 2014a). The nine sessions comprised six NFT sessions and three generalization sessions. In the NFT sessions, participants received real-time visual feedback of their own brain activity. In the generalization sessions, participants were instructed to achieve the mental states as in the NFT sessions, but they did not receive feedback on their actual brain activity. These generalization sessions were used to investigate whether participants are able to transfer mental states achieved during feedback conditions to situations without feedback (Gruzelier, 2014b). We also included a control group receiving feedback on random EEG frequencies. Another central feature of the present design is the almost complete removal of social reinforcement from the training protocol. Textbooks and review articles on NF underestimate the role of psychosocial factors in driving NF success (Thibault and Raz, 2016; Thibault et al., 2017; Wood and Kober, 2018). Participants only received instructions in person once at the beginning of training, on the occasion of handing out the equipment for home-based training. Therefore, positive training effects cannot be attributed to the motivating role of the experimenter or NF trainer/therapist (Chapman et al., 2018). Additionally, we used a double-blind design to rule out possible experimenter effects.

We also assessed participants’ individual mental strategies during the NFT to investigate, on the one hand, whether mental strategies differ between the experimental group (receiving feedback of SMR) and the control group (receiving feedback of random EEG frequencies), and on the other hand to qualitatively compare mental strategies reported in a home-based NF setting with mental strategies reported in prior studies performed in a lab environment (Kober et al., 2013, 2017; Autenrieth et al., 2020).

## 2. Materials and methods

### 2.1. Participants

A total of 38 healthy young adults (19 women) participated in this study. Participants were assigned to one of two NFT groups: A SMR up-regulation group (9 males, 10 females, mean age = 23.89 years, *SD* age = 2.65) and a control group (10 males, 9 females, mean age = 25.21 years, *SD* age = 4.49) that performed NFT sessions with feedback in a randomly selected EEG frequency range. Volunteers were blind to the grouping design and did not know that there were different conditions. All participants fulfilled the following inclusion criteria at the start of the study: (1) no neurological or psychological disorders, (2) no severe diseases, (3) no symptoms that interfere with recording biosignals (e.g., skin problems, wounds in the head area, uncontrolled muscle movements), (4) no medication that affects the central nervous system, (5) no Reflex Epilepsy, and (6) no prior experience with neurofeedback. The study was approved by the local Ethics Committee of the University of Graz, Austria (GZ. 39/48/63 ex 2020/21) and is in accordance with The Code of Ethics of the World Medical Association (Declaration of Helsinki) for experiments involving humans (WMA World Medical Association, 2009). All volunteers gave written informed consent and received for their participation either research credit hours (9 h in total) for their Psychology Bachelor program or money (72€ in total).

After completion of the study, 11 subjects (5 women) had to be excluded from the sample due to technical problems (NF software did not save the required markers for the runs and therefore these sessions could not be divided in the appropriate segments). Additionally, two subjects (one woman) had to be excluded because of non-compliance with the instructions or the schedule. Therefore, we analyzed the EEG data of 25 subjects (13 women). After EEG analysis, 8 participants had to be excluded from the sample because they did not match the EEG data quality criterion (see “EEG data recording and analysis” for details). In the final sample of *n* = 17 subjects, *n* = 8 subjects (7 women, mean age = 23.40 years, *SD* age = 1.50 years) were assigned to the experimental group and *n* = 9 subjects (4 women, mean age = 23.70 years, *SD* age = 2.87 years) were assigned to the control group.

### 2.2. Procedure

**FIGURE 1:**
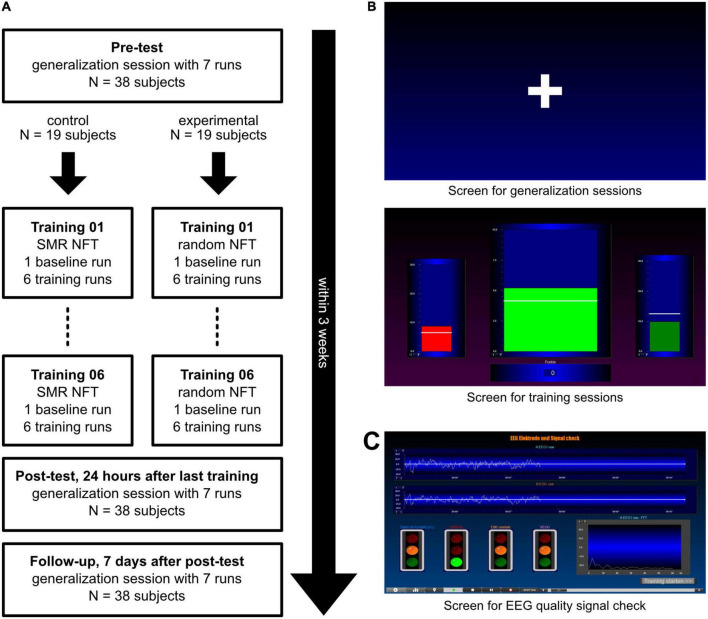
Procedure and illustration of the presented screens. **(A)** After a pre-test, subjects were split into two groups: The experimental group received 6 sensorimotor rhythm (SMR) neurofeedback training (NFT) sessions while the control group performed six NFT sessions with feedback in a random 4 Hz frequency range between 7 and 20 Hz. After the NFT sessions, every subject performed two generalization sessions. **(B)** In the generalization sessions (top panel), participants saw a white fixation cross in the center of the screen. In the training sessions (bottom panel), three moving bars were displayed: Theta (left bar, 4–7 Hz), the frequency to be trained (middle bar; SMR, 12–15 Hz, in the experimental group, and a random 4 Hz frequency band in the control group) and Beta (right bar, 21–35 Hz). White lines depict individual thresholds. **(C)** The EEG data quality signal check was displayed before the start of every session and showed the biosignals of the EEG and EOG electrodes (top) as well as a fast fourier transformation (FFT) graph to detect 50 Hz artifacts (bottom right).

This study used a double-blind pre-post design with a follow-up measurement and consisted of 9 sessions (Figure 1A). Each session was conducted on different days independently by the participants at home without the attendance of an experimenter. All subjects performed six NFT sessions within 2 weeks. Before the first (pre-test) and after the last post-test) NFT session, subjects performed a generalization session. They should try to achieve the desired mental state from the NFT without any feedback being displayed. The follow-up measurement was another generalization session 7 days after the post-test. After each session, participants had to answer four questions about their subjective experience during the session on Visual Analog Scales (VAS). The questions were: (1) “How strongly are your thoughts focused on the neurofeedback task?”, (2) “How well can you concentrate on the neurofeedback task compared to your regular concentration?”, (3) “How successful would you rate your session today?”, and (4) “How satisfied are you with your performance today?” Participants were also asked to describe their mental strategies after the first, second, seventh, eighth, and last session (Kober et al., 2013, 2017; Autenrieth et al., 2020). The following instructions were presented to all participants: *“Please describe in your own words the strategies you used during neurofeedback training to control the bars.”* (in NFT sessions) and *“Please describe in your own words what strategies you used in this session.”* (in generalization sessions).

**FIGURE 2:**
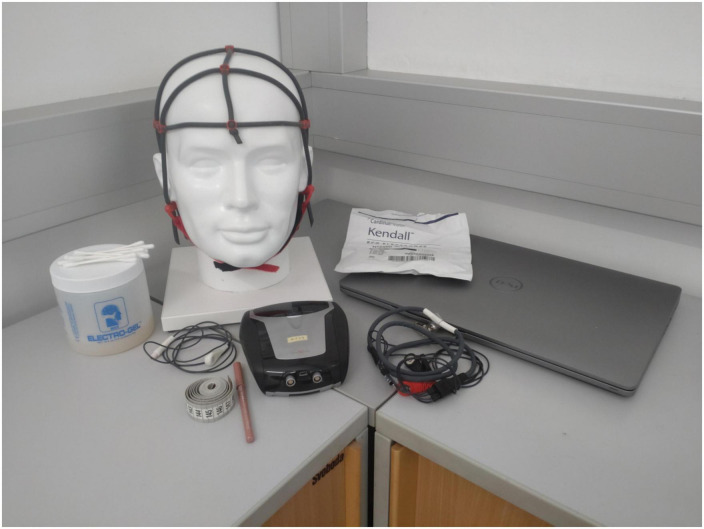
Illustration of the provided home-based neurofeedback (NF) system.

Two female experimenters conducted the study. Interactions between participants and experimenters were limited to solving technical problems, organizing schedules, and sending reminders. Subjects could contact the experimenters anytime via email or mobile phone (text messages or calls), but not in person. The experimenters sent text reminders to the participants on the days of their sessions, but they were not present during the sessions. At the first meeting, the experimenter explained the EEG system, handed it over, and determined the individual schedule with the participant. The EEG system included a Portable 10-channel EEG amplifier (NeXus-10 MKII, Mind Media BV, Herten, Netherlands), a Lenovo laptop on which the protocols were running (BioTrace + software, Mind Media BV, Herten, Netherlands), EEG disks, and EMG/ECG adhesive electrodes for measuring EEG signal and eye movements, respectively, a cap to fixate electrodes on the head, a water-soluble colored pen to help marking the exact position of the EEG electrode on the participant’s head, electrogel for the EEG electrode, a measuring tape, and consumable materials, i.e., cotton swabs/pads and alcohol for disinfecting skin areas (Figure 2). Subjects received written instructions with pictures for the following points: (1) mounting the EEG system and cleaning it after usage, (2) connecting the amplifier with the laptop *via* Bluetooth, (3) starting the NFT program, (4) checking the EEG signal quality, and (5) saving the session data. There was also a video instruction available on how to mount the EEG system and clean it after usage. There was no demonstration of the montage of the EEG system or the NFT program performed by the experimenters, but the participants received the hint to use a mirror or the front camera of the laptop during the EEG montage. Due to the detailed illustrated instructions, no special PC knowledge was required.

After completion of the study, participants were informed about their group assignment. In order to maintain double blindness, this meeting was conducted by an independent person. The experimenters were informed about the group assignments after conclusion of all measurements and the statistical analysis.

### 2.3. Neurofeedback training and generalization sessions

The paradigms for the sessions were generated with the BioTrace + software (Mind Media BV, Herten, Netherlands). The instructions for the sessions were implemented in the training program and were presented before the start of every session.

During the NFT sessions, participants saw three bars moving up and down (Figure 1B, bottom panel). In the experimental group, the central bar showed SMR activity (12–15 Hz), whereas, in the control group, the central bar reflected the EEG activity of a random 4 Hz frequency band in a range of 7–20 Hz (excluding 12–15 Hz). In the control group, the feedback frequency band changed at each of the six NFT sessions. The order of each frequency band used was identical for all subjects: 9–12, 15–18, 7–10, 16–19, 17–20, and 8–11 Hz. The two lateral bars were used to control eye and muscle movements. Eye movements are commonly associated with increases in slower frequencies (e.g., Theta, 4–7 Hz), whereas muscle activity is associated with increases in faster frequencies (e.g., Beta, 21–35 Hz). These artifacts can lead to an increase in SMR activity. To prevent participants from falsely increasing their SMR activity using eye and muscle movements (e.g., excessive blinking or conscious muscle tensing), Theta activity (4–7 Hz) was presented in the left bar, and Beta activity (21–35 Hz) was displayed in the right bar in both groups (Doppelmayr and Weber, 2011; Weber et al., 2011).

Each NFT session consisted of seven runs of 3 min each. The first run was a baseline run, in which the participant’s task was to observe the movement of the bars. This baseline was necessary to calculate individual thresholds displayed on the screen as white horizontal lines in the subsequent feedback runs. The thresholds for the two lateral bars were constant in all six training runs and displayed the average EEG power in these frequencies as observed during the baseline run +1 SD. The central bar’s threshold depicted the average SMR (or the random EEG frequency in the control group) power observed during the baseline and previous runs. That is, the thresholds were adapted to the level of the individuals in each training run.

Participants were instructed to increase the central bar’s size and to keep the size of the two lateral bars constantly below their thresholds. Whenever the size of the central bar reached the individual threshold, and simultaneously the sizes of the two lateral bars were kept below their respective thresholds, the subjects were rewarded with positive feedback. The color of the central bar switched from red to green, auditory feedback was given in the form of a “Pling” sound, and the participants received points (depicted under the bar in the middle of the screen, Figure 1B). The goal was to achieve as many points as possible. The participants were instructed to stay relaxed and focused at the same time.

During the generalization sessions, participants saw a white fixation cross in the center of the screen (Figure 1B, top panel). Each session also consisted of 7 runs of 3 min each. In the pre-test, participants were instructed to look at the fixation cross and try to achieve a mental state where they were relaxed and focused simultaneously. In the post-test and follow-up, participants were instructed to achieve the desired mental state from the NFT sessions without visual and auditory feedback.

We summarized our study’s reporting and experimental design according to the CRED-nf best practices checklist (Ros et al., 2020, Supplementary material A).

### 2.4. EEG data recording and analysis

Electroencephalogram recording was performed using a NeXus MKII-10 amplifier (MindMedia BV, Herten, Netherlands), and the signal was digitized at 256 Hz. The EEG electrode was placed on the Cz position (according to the international 10–20 EEG placement system), the ground was placed at the right mastoid, and the reference electrode was located at the left mastoid. Additionally, one EOG channel was recorded. Therefore, one electrode was placed above, and the reference electrode was placed below the left eye. Participants reported no difficulties placing the electrodes on their heads, starting the EEG measurements, and independently performing the training or generalization sessions.

After data collection, EEG datasets were analyzed offline using BrainVision Analyzer software (version 2.2, BrainProducts GmbH, Munich, Germany). First, a semi-automatic artifact correction was performed. The following exclusion criteria were set: (1) >50 μV voltage difference between two data points, (2) >200 μV voltage difference within a 200 ms interval, and (3) absolute voltage values ±120 μV. Afterward, all remaining artifacts (e.g., muscle movements or noisy signals) were manually removed by visual inspection of the data. An adaptation of the standard artifact correction criteria was necessary for some data sets due to the specifics of the individual EEG signal. Thus, a notch filter (50 Hz) was applied to ten participants before semi-automatic artifact correction. Furthermore, due to unusually large amplitudes in the EOG channel, this channel of two participants was examined at a larger μV range (200 μV instead of the usual 100 μV) during the manual inspection to detect eye movements successfully (e.g., blinks) despite the high amplitudes. All 1-s epochs with artifacts were excluded from the EEG analysis (about 25% of the data from the final sample). For the EEG analysis of the experimental and control group, absolute SMR (12–15 Hz), Theta (4–7 Hz), and Beta (21–35 Hz) band power were extracted using complex demodulation (Brain Products GmbH, 2009). The extracted power values were averaged over the whole artifact-free runs in one session. Visual inspection showed a right-skewed distribution. Therefore, absolute SMR, Theta, and Beta power values were log-transformed before statistical analyses were performed.

After EEG analysis, participants were excluded from the sample, if they did not match the following EEG data quality criterion: a minimum of 4/7 usable runs (≥80/180 artifact-free segments) per session, and a minimum of 6/9 usable sessions per subject.

### 2.5. Statistical analysis

To evaluate the within- and between-session changes in EEG power simultaneously, we employed two separate mixed-effects models to analyze the training and generalization sessions. The use of two separate models was necessary to ascertain homoscedasticity. For the first model (training sessions), we used the linear fixed effects group (experimental vs. control group), session (early = sessions 1–3 vs. late = sessions 4–6), and run (early = baseline + run 1–3 vs. late = run 4–6) as a triple interaction for the dependent variable SMR power (Type I Analysis of Variance with Satterthwaite’s method). We included session (early vs. late) as a by-subject random slope. The second model (generalization sessions) included the same variables including session as a by-subject random slope, but here we defined the linear fixed effect session differently (pre = generalization session before first training vs. post = both generalization sessions after last training). The use of dichotomous variables (session and run) was necessary to avoid convergence problems. The development and calculation of the mixed-effects models can be found in Supplementary material B. We calculated multiple pairwise comparisons to analyze significant interactions, including Bonferroni correction for multiple testing.

Additionally, we performed frequency analysis with the reported mental strategies. For this purpose, the statements are segmented into tokens (single words) and cleaned from stopwords (function words with little to no substantive meaning for the content, e.g., and, but, if, …). The remaining words can then be counted to make statements about the frequencies of their occurrence. We analyzed the answers of 25 subjects (17 subjects + 8 subjects excluded due to their bad EEG quality). This study was conducted in German, therefore the frequency analysis also refers to the German words. The results table was translated into English for the report, which is why some direct translations are not always straightforward.

Lastly, we examined the success of blinding for the complete sample of *n* = 38 participants. Therefore, we performed a Chi-squared test with Yates’ continuity correction because expected counts in half of the cells were <10.

All statistical analyses were performed in R 3.4.1 (R Core Team, 2022). For mixed-effects modeling, the *lme4* package was used (Bates et al., 2015), and for the frequency analysis of the mental strategies, the package *quanteda* (Benoit et al., 2018) was used. Pairwise comparisons for significant interactions were calculated using the package *emmeans* (Lenth, 2022). Alpha level was set to *p* = 0.05. R Code can be found in Supplementary material B.

## 3. Results

### 3.1. Success of blinding

At the end of the study, 63% of the participants could successfully guess which group they had been assigned to (experimental group: 53% or 10/19 correct answers; control group: 74% or 14/19 correct answers). A Chi-squared test with Yates’ continuity correction revealed no significant difference between the two groups, *X*^2^(1, 38) = 1.02, *p* = 0.31, which is why independence between the group assignments can be assumed, and the blinding of the participants can be classified as successful.

### 3.2. NF performance

The results of the mixed-effects models for the dependent variable SMR power are presented in Table 1, separately for the training and generalization sessions.

**TABLE 1:** Results of the mixed-effects models with the linear effects group (experimental or control group), session (early vs. late sessions), and run (early vs. late runs), and the by-subject random slope session (early vs. late sessions) for the dependent variable log-transformed sensorimotor rhythm (SMR) power, presented separately for the training and generalization sessions.

		NFT sessions			Generalization sessions		
		*F* (df, df error)	MSE	*P*-value *(η^2^_*p*_)*	*F* (df, df error)	MSE	*P*-value *(η^2^_*p*_)*
log-SMR power	Group	1.70 (1, 17.02)	0.081	0.21	4.52 (1, 17.21)	0.334	0.048* (0.015)
	Session	0.13 (1, 16.94)	0.006	0.73	0.35 (1, 16.22)	0.026	0.56
	Run	0.07 (1, 609.48)	0.003	0.80	8.66 (1, 284.65)	0.641	0.004** (0.029)
	Group * Session	0.45 (1, 16.96)	0.021	0.51	4.57 (1, 16.20)	0.338	0.048* (0.016)
	Group * Run	0.21 (1, 609.21)	0.010	0.65	2.47 (1, 284.61)	0.183	0.12
	Session * Run	1.17 (1, 609.22)	0.056	0.28	0.75 (1, 284.39)	0.055	0.39
	Group * Session * Run	1.31 (1, 609.22)	0.063	0.25	0.90 (1, 284.39)	0.067	0.34

Significant results are marked with *. (*p < 0.05, **p < 0.01).

**FIGURE 3:**
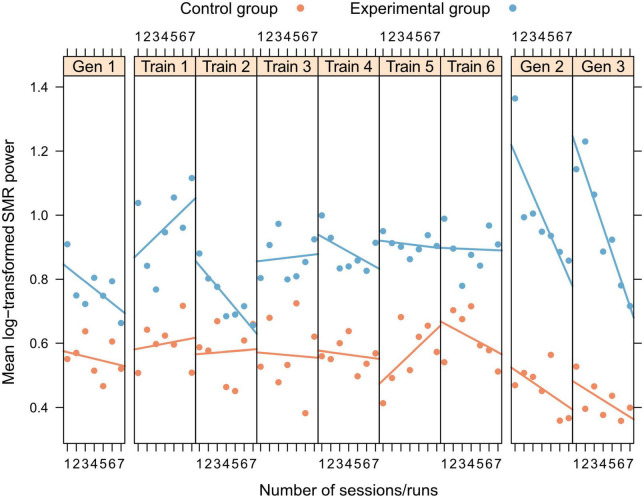
Within- and between-session changes in log-transformed SMR power for training and generalization sessions, separately presented for experimental (blue) and control group (orange).

Neurofeedback training: No significant changes in SMR power within and across NFT sessions were shown by any of the two groups (all *p* > 0.21, Figure 3).

Generalization sessions: The experimental group showed a significantly higher SMR power during generalization sessions than the control group (significant main effect group, *n^2^_*p*_* = 0.015, Figure 3, Table 2). There were no significant changes between generalization sessions, but there was a significant main effect run, which can be seen in a significant decrease of SMR power within generalization sessions (*n^2^_*p*_* = 0.029, Figure 3). Table 3 shows within-session changes in SMR power, separately for the experimental and control group. Additionally, there was a significant interaction group × session, which could not be confirmed by post-tests (*n^2^_*p*_* = 0.016, all pairwise comparisons: *p* > 0.07).

**TABLE 2:** Between session changes in mean log-transformed SMR, Theta, and Beta power, presented separately for the experimental and control group with Cosineau-Morey transformed standard errors.

	Experimental group			Control group		
	Mean log-power *(SE)*			Mean log-power *(SE)*		
	SMR	Theta	Beta	SMR	Theta	Beta
Gen 1	0.77 (0.041)	1.75 (0.057)	1.35 (0.041)	0.55 (0.025)	1.63 (0.015)	1.22 (0.039)
Train 1	0.96 (0.041)	1.67 (0.044)	1.58 (0.066)	0.60 (0.037)	1.68 (0.025)	1.37 (0.043)
Train 2	0.74 (0.035)	1.69 (0.030)	1.25 (0.035)	0.58 (0.038)	1.74 (0.021)	1.26 (0.053)
Train 3	0.87 (0.037)	1.71 (0.043)	1.50 (0.045)	0.57 (0.044)	1.60 (0.046)	1.56 (0.074)
Train 4	0.88 (0.043)	1.81 (0.047)	1.23 (0.039)	0.57 (0.023)	1.76 (0.022)	1.45 (0.046)
Train 5	0.91 (0.028)	1.82 (0.041)	1.32 (0.043)	0.58 (0.026)	1.70 (0.019)	1.40 (0.049)
Train 6	0.89 (0.031)	1.71 (0.043)	1.23 (0.040)	0.61 (0.040)	1.79 (0.035)	1.25 (0.054)
Gen 2	0.99 (0.049)	1.88 (0.069)	1.15 (0.035)	0.46 (0.043)	1.58 (0.027)	1.05 (0.069)
Gen 3	0.97 (0.086)	1.98 (0.099)	1.46 (0.072)	0.42 (0.039)	1.62 (0.042)	1.06 (0.065)

**TABLE 3:** Within session changes (averaged across seven runs per session) in mean log-transformed SMR, Theta and Beta power, presented separately for the experimental and control group with Cosineau-Morey transformed standard errors.

	Experimental group			Control group		
	Mean log-power *(SE)*			Mean log-power *(SE)*		
	SMR	Theta	Beta	SMR	Theta	Beta
Baseline	0.99 (0.051)	1.77 (0.052)	1.36 (0.043)	0.52 (0.025)	1.62 (0.029)	1.22 (0.038)
Run 1	0.92 (0.052)	1.80 (0.057)	1.35 (0.052)	0.57 (0.033)	1.70 (0.027)	1.32 (0.047)
Run 2	0.88 (0.044)	1.79 (0.058)	1.35 (0.046)	0.59 (0.036)	1.72 (0.025)	1.33 (0.050)
Run 3	0.84 (0.039)	1.81 (0.054)	1.33 (0.038)	0.54 (0.032)	1.70 (0.035)	1.25 (0.051)
Run 4	0.86 (0.040)	1.79 (0.054)	1.36 (0.046)	0.55 (0.032)	1.67 (0.025)	1.28 (0.051)
Run 5	0.86 (0.032)	1.76 (0.041)	1.33 (0.046)	0.55 (0.038)	1.68 (0.024)	1.31 (0.062)
Run 6	0.84 (0.036)	1.75 (0.044)	1.31 (0.039)	0.52 (0.033)	1.69 (0.026)	1.28 (0.064)

Additionally, results of the mixed-effects models, separately for the dependent variables Theta and Beta power, are presented in Supplementary material C.

### 3.3. Mental strategies

A frequency analysis showed that the experimental group used 106 different words across all sample points, and the control group used 87 different words. Interestingly, only 10–21 words or 9–13 words were used by more than one participant in the experimental or control group, respectively.

The most frequently used word across all sample points in both groups was the word “bar” (experimental group: 31 times; control group: 24 times). Table 4 lists the top 5 words of both groups, ranked by the percentage of users, and separated by the five sample points. Interestingly, both groups showed a similar choice of words: The word “bar” was the most frequently used word by both groups in the first and last NFT sessions. Similar words were also used at the other time points. For example, both groups used the words “tried,” “cross,” and “focus” more frequently in the generalization sessions than in the NFT sessions. In summary, this reveals a similar semantic structure of the mental strategies in the two groups.

**TABLE 4:** Top five words, ranked by the percentage of users, separately for the experimental and control group.

	Experimental group	Control group
Gen 1	tried (36%), cross (36%), possible (29%), time (14%), and always (14%)	cross (27%), tried (27%), count (27%), think (27%), and concentrate (27%)
Train 1	bar (57%), tried (29%), middle (36%), eyes (21%), and points (21%)	bar (45%), thought (27%), middle (27%), tried (18%), and count (18%)
Train 6	bar (50%), tried (29%), middle (29%), always (21%), and calm (21%)	bar (45%), tried (27%), concentrate (27%), think (18%), and points (18%)
Gen 2	concentrate (43%), bar (36%), tried (29%), cross (29%), and calm (21%)	tried (45%), thoughts (36%), bar (18%), think (18%), and thought (18%)
Gen 3	tried (36%), bar (29%), cross (29%), sessions (21%), and fell* (21%)	tried (36%), bar (27%), remember (18%), and point (18%)

In this table, mental strategies were translated from German to English. *“fell” (“fiel” in German) means in this context “it was easy for me” (“es fiel mir leicht” in German).

## 4. Discussion

In the present study, we assessed the capacity of healthy individuals to modulate their own SMR when using a home-based NFT system on their own for 3 weeks. Based on the increasing number of commercially available home-based NFT systems, which are not remotely supervised, personally instructed or guided by a NF trainer, therapist or experimenter, we wanted to test whether participants can perform an EEG-based NFT on their own at their home. Therefore, we kept participants’ face-to-face contact with experimenters to a minimum, and instructions were provided only in the form of written information or videos. We included an active control group receiving feedback of random EEG frequencies to be able to differentiate between NFT specific and unspecific effects (Thibault et al., 2017; Ros et al., 2020). Additionally, we wanted to control for expectation and experimenter effects by using a double-blind design.

In contrast to previous SMR-based NFT studies performed in a lab environment with personal interaction with a NF instructor or experimenter (Kober et al., 2013, 2015, 2018, 2020; Gruzelier, 2014a; Autenrieth et al., 2020), participants were not able to voluntarily increase their SMR during NFT. We neither found within nor between session changes in SMR activity in either group. This might be due to different reasons.

First, the personal interaction with a NF expert might be of relevance. In the present study, social interaction between trainer and participant were reduced to a minimum: participants received instructions in person only once at the beginning of the study, when equipment was handed to individual participants. Beyond this first contact, instructions on turning on and mounting NF equipment at home was given as an instructional video, which could be watched from home as often as desired. The absence of both training outcome and trainer-learner-interaction indicates that learning to modulate one’s own brain activity includes more than having access to a NFT system and knowing the theoretical aspects of the technique. Glannon (2014) describes NFT as a biopsychosocial process, where the NF trainer plays a crucial role in effecting the training outcome. One may even describe this trainer-learner-interaction as some sort of doctor-patient relationship. The importance of this relationship is addressed more extensively in medicine (see Benedetti, 2011) but should also be more considered in NFT settings, especially when NF is used as a therapeutic intervention. For example, coming to a lab or a clinic might increase participants’ adherence to or compliance with the training. Compliance to treatment is an important topic in NFT as it is generally in the realm of TR. Therefore, the evidence for effects of NFT establish a lower-bound on NFT efficacy, which is observed when opportunities of social contact and social reinforcement are removed from the learning protocol almost completely. In this context, some NFT studies provide evidence that psychosocial factors play a crucial role in NF success (Wood and Kober, 2018).

Second, a remote supervision of the training using e.g., a video chat function might be necessary to control EEG data quality, keep participants motivated, remind them of the training schedule and give guidance on EEG montage, etc. In a previous home-based NFT study, in which patients with Multiple Sclerosis performed SMR-based NFT at home on their own, half of the patients were able to linearly increase SMR activity during NFT (Kober et al., 2019). In this study, a therapist system was used to enable the NF trainer to monitor the NFT and EEG data quality remotely while participants performed the NFT at home. Kober et al. (2019) also used a video chat function to communicate with the participants during the NFT. Additionally, the NF trainer went to the patients’ home before the first NFT session to instruct and train the patients and to prepare the NF setup. In another home-based NFT study, patients with Insomnia performed 20 sessions to upregulate their SMR (Cortoos et al., 2010). Here, participants received an explanation and hands-on training for the training sessions at home. In every training session, participants were called by the therapist. The therapist initiated the training program and checked the EEG signal quality, then the subjects performed the session on their own. These two examples are in marked contrast to the present study, which was conducted in a comparable manner as if participants had purchased a commercially available, unsupervised home-based NF system. This strongly indicates that a remote supervision of the training and a more direct communication with the NF users are necessary to enable successful NFT.

Third, the EEG data quality of the present study was not as good as in previous studies performed in the lab. Here, we had to exclude eight of 25 participants due to bad EEG data quality. Additionally, of the remaining data sets, 25% of the EEG data was excluded due to artifacts. Prior SMR-based NF studies using the same EEG equipment and training software performed in a lab environment report on about 9–15% data exclusion due to artifacts (e.g., Kober et al., 2013, 2020). The large loss of data in the present study could again be explained by the need for remote monitoring of NFT. Although participants did not report any problems with the EEG montage and the implementation of the NFT, a large amount of data had to be excluded afterward. This indicates that participants with no prior NF experience had problems in judging EEG data quality during training. In the present study, subjects had to check the EEG data quality with a signal check screen implemented in the training protocol (see Figure 1C). One participant reported after completion of the study that the signal check screen was confusing, which is why the design of this screen could be adapted to be more appropriate for EEG novices. Additionally, the signal check screen was only displayed once before the baseline run, therefore a repeated signal check after every run could help increase the EEG data quality as well.

We also included generalization sessions, in which participants should reach comparable mental states as during NFT, although they did not get any feedback of their actual brain activity. The inclusion of such generalization sessions is a valuable tool to determine whether NF users are able to generalize or transfer mental states achieved during NF training, such as those associated with improved cognitive performance (Gruzelier, 2014b; Kober et al., 2015) to other contexts. However, since participants did not learn how to control their SMR activity they also did not show meaningful or training specific changes in SMR activity during these generalization sessions. The experimental group showed an overall higher SMR power during the generalization sessions than the control group. Our sample size per group was not very large after data exclusion. Therefore, interindividual differences in absolute SMR amplitude may vary randomly between groups. SMR power also decreased over the runs within the generalization sessions. This might be a general effect of a decrease in attentional focus or increase in tiredness over time. Participants received the instruction of being mentally focused but physically relaxed at the same time, since SMR emerges during such a state (Sterman, 1996, 2000; Serruya and Kahana, 2008; Gruzelier et al., 2010). It is likely that participants in both groups were unable to maintain this mental state during the generalization session, so declining concentration and mental focus over time may have caused the decrease in SMR power. The inability to maintain the desired mental state may also result from the absence of feedback during the generalization sessions. If there is no feedback, participants can only rely on their subjective experience that they encountered during the training through positive feedback. According to Davelaar (2018), this subjective experience can become the feedback signal in form of a secondary reinforcer. To achieve this, NF learners have to pair their subjective experiences with the reward signal. We can assume, that this pairing was not successful in our participants, because they did not show training specific changes in SMR within and between the training sessions. Consequently, if there is no formed secondary reinforcer, it is almost impossible to maintain the desired mental state without feedback, so this may have also caused the decrease in SMR power. Exploratively, mental strategies used during NF training as well as during the generalization sessions were assessed. The experimental group used more different words to describe the mental strategies they used than the control group. Only a relatively small number of words was used by more than one participant in both groups. Generally, successful SMR NF performance is associated with reporting no specific strategy (Kober et al., 2013, 2017). Hence, the finding that the experimental group used even more different words to describe their mental strategies than the control group could be a sign of the non-functioning of the NFT. Although NFT was not successful in the present study, the words reported by the participants did not differ to reports on mental strategies assessed in laboratory settings (Kober et al., 2013, 2017; Autenrieth et al., 2020). In previous studies, words were classified into different categories (Kober et al., 2013, 2017; Autenrieth et al., 2020). Mentioning the feedback bar for instance was commonly reported in previous NF studies and was classified as visual strategy (Autenrieth et al., 2020). Interestingly, the mental strategies are quite similar between the NFT sessions and the generalization sessions. “Bar” is also frequently mentioned in the generalization sessions, although no feedback bar was presented in this condition. Since participants were not successful in up-regulating their SMR activity during NF training, the assessed mental strategies say nothing about successful NF strategies, but merely indicate that participants were focused on the NF task and read the instructions.

### 4.1. Limitations

There are a few limitations to the present study that need to be considered. The small final sample size (*n* = 17) affects the power of our results, which should be taken into account when interpreting our results. As already pointed out, the overall higher SMR power in the experimental group in the generalization sessions may have been caused by chance due to the small sample size. Additionally, we did not control for possible gender differences in our final sample. Although we ensured a balanced gender proportion in both groups in our original sample (*n* = 38, 19 women), the final sample (11 women) was no longer gender balanced due to a variety of exclusions. Lastly, because our participants trained at home on their own, we could not control their environments. We did not record how focused the individuals were during the training sessions and how much attention they paid to the instructions. This is a common disadvantage of such home protocols, but at the same time allows a more realistic implementation as commonly seen in commercially available NFT systems.

For the future, home-based NF studies should include a group conducting the NF sessions in a laboratory environment. In doing so, missing NF learning performance could be associated with the absence of social interactions between NF trainer and NF user regardless of the training environment. These future studies should also ensure a larger final sample size.

## 5. Conclusion and future directions

To summarize, the results of the home-based NFT strongly suggest the need for personal interaction and instructions between the NF users and the NF trainer. In the future, technologies should be developed to support the establishment of adequate training conditions at home. With the help of smartphone sensors, levels of noise and presence of distractions can be easily controlled by means of a dedicated app. Moreover, the quality of EEG montage and channel signals could also be made more straightforward to lay persons. A substantial number of participants had to be excluded because of software error. The technology readiness level of existing systems is already high enough to generate positive training effects but since the majority of NFT systems was not conceived with fully unsupervised home-based training in mind, some further improvements have to be added to existing systems for them to fully support home-based NFT. For example, a signal quality check should be implemented in such a way that it can be performed quickly and without any major possible misinterpretations, even by lay persons. The rate of possible misinterpretations of the signal could be reduced if the quality check is automatically performed by the NFT program and just informs the NF user if he/she needs to adjust the electrodes. In an automatized implementation, the signal check could also be done continuously during the training to ensure good data quality over the entire duration of the recording. To ensure that the NF user focus on the NFT itself, cameras (e.g., the webcam of a notebook) could be used as an eye tracker to check if the NF user keeps his/her eyes open and looks directly at the screen to avoid unintentional manipulation of the EEG signal which consequently would manipulate the training outcome.

## Data availability statement

The original contributions presented in this study are included in the article/Supplementary material, further inquiries can be directed to the corresponding author.

## Ethics statement

The studies involving human participants were reviewed and approved by the Ethics Committee of the University of Graz under GZ. 39/48/63 ex 2020/21. The patients/participants provided their written informed consent to participate in this study.

## Author contributions

MA organized and conducted the study. SK and MA analyzed EEG data. MA and GW did the statistical analysis. All authors reviewed, edited, approved, and wrote the manuscript.
