# Supplementary material for: Assessment of the capacity to modulate brain signals in a home-based SMR neurofeedback training setting
Source: Front Hum Neurosci. 2023 Jan 5;16:1032222. doi: 10.3389/fnhum.2022.1032222 (PMC9849904; doi:10.3389/fnhum.2022.1032222)
Supplement: Supplementary file 1 [file Data_Sheet_1.pdf]

Supplementary Material A

Consensus on the reporting and experimental design of clinical and cognitive-behavioural neurofeedback studies (CRED-nf checklist) best practices checklist (Ros et al., 2020).

Domain	Item #	Checklist item	Reported in	Comments
**Pre-experiment**				
	1a	Pre-register experimental protocol and planned analyses	X	This was an explorative study.
	1b	Justify sample size	X	This was an explorative study.
	2a	Employ control group(s) or control condition(s)	Methods section	Active control group
	2b	When leveraging experimental designs where a double-blind is possible, use a double-blind	Methods section	Participants did not know whether they received feedback on their SMR or not. Experimenters did not know group assignments until statistical analysis were finished.
	2c	Blind those who rate the outcomes, and when possible, the statisticians involved	Methods section	Experimenters were informed about group assignments after the completion of the statistical analysis.
	2d	Examine to what extent participants and experimenters remain blinded	Results section	Participants were asked about their group assignment after completion of the study. The success of blinding was examined using a Chi-squared test.
	2e	In clinical efficacy studies, employ a standard-of-care intervention group as a benchmark for improvement	X	This was not a clinical efficacy study.
	3a	Collect data on psychosocial factors	Methods section	Participants were asked every session about their subjective experiences.
	3b	Report whether participants were provided with a strategy	Methods sections	
	3c	Report the strategies participants used	Results section	
	3d	Report methods used for online-data processing and artifact correction	Methods section	
	3e	Report condition and group effects for artifacts		

	4a	Report how the online-feature extraction was defined	Method section	
	4b	Report and justify the reinforcement schedule	Method section	
	4c	Report the feedback modality and content	Method section	
	4d	Collect and report all brain activity variable(s) and/or contrasts used for feedback, as displayed to experimental participants	Method section, Results section & Supplementary Material C	
	4e	Report the hardware and software used	Method section	
Brain	5a	Report neurofeedback regulation success based on the feedback signal	Results section	
	5b	Plot within-session and between-session regulation blocks of feedback variable(s), as well as pre-to-post resting baselines or contrasts	Results section	
	5c	Statistically compare the experimental condition/group to the control condition(s)/group(s) (not only each group to baseline measures)	Results section	
Behaviour	6a	Include measures of clinical or behavioural significance, defined a priori, and describe whether they were reached	X	
	6b	Run correlational analyses between regulation success and behavioural outcomes	X	
**Data storage**				
	7a	Upload all materials, analysis scripts, code, and raw data used for analyses, as well as final values, to an open access data repository, when feasible	See data availability	

Literature

Ros, T., Enriquez-Geppert, S., Zotev, V., Young, K. D., Wood, G., Whitfield-Gabrieli, S., ... & Thibault, R. T. (2020). Consensus on the reporting and experimental design of clinical and cognitive-behavioural neurofeedback studies (CRED-nf checklist).
