# Supplementary material for: Assessment of the capacity to modulate brain signals in a home-based SMR neurofeedback training setting
Source: Front Hum Neurosci. 2023 Jan 5;16:1032222. doi: 10.3389/fnhum.2022.1032222 (PMC9849904; doi:10.3389/fnhum.2022.1032222)
Supplement: Supplementary file 2 [file Data_Sheet_2.pdf]

Supplementary Material B: R Code for statistical analysis

This RMarkdown shows the development of the linear mixed-effects models for the training + generalization sessions, and the frequency analysis of the mental strategies.

```
library(tidyverse) # for cleaning up data
library(readxl) # for importing excel sheet
library(lme4) # for calculating linear mixed-effects models
library(emmeans) # for performing post-tests for sign. interactions
library(lmerTest) # for anova and p value
library(dlookr) # for checking distribution of the dataset

library(sjstats) # for anova + partial eta squared; please note: originally,
# the function anova_stats(model) can only calculate Type III Sum of Squares,
# therefore we forked the package and changed the source code, so that
# Type I Sum of Squares can be calculated. If interested, install
# changed package with the following line:
# devtools::install_github("mautenr/sjstats")

# for frequency analysis (mental strategies)
library(quanteda)
library(quanteda.textmodels)
library(quanteda.textstats)
library(quanteda.textplots)
```

```
data <- as_tibble(read.csv("eeg_data.csv", sep = ";", na.strings = "NA"))

# Converting variables in their desired data type
data$session_n <- as.numeric(data$session_n)
data$run_n <- as.numeric(data$run_n)

# defining new variable: Training or generalization as dichotomous variable
data$NForGEN <- NA
data$NForGEN[data$session_n %in% c(2:7)] <- 1 # training
data$NForGEN[data$session_n %in% c(1, 8, 9)] <- 2 # generalization
data$NForGEN <- as.factor(data$NForGEN)

head(data) # sex: 0 = female, 1 = male; eeg-power: absolute power-values
```

Loading R packages and importing dataset

```
## # A tibble: 6 x 9
```

```
##   subject group     sex   age session_n run_n frequency eeg_power NForGEN
##   <chr>   <chr>   <int> <int>     <dbl> <dbl> <chr>         <dbl> <fct>
## 1 p02     Control     0    25         1     1 Beta           7.64 2
## 2 p02     Control     0    25         1     2 Beta           6.85 2
## 3 p02     Control     0    25         1     3 Beta          NA    2
## 4 p02     Control     0    25         1     4 Beta           5.30 2
## 5 p02     Control     0    25         1     5 Beta           6.23 2
## 6 p02     Control     0    25         1     6 Beta          NA    2
```

```
# subsetting dataset into training and generalization sessions for SMR
data_smr <- subset(data, frequency == "SMR")
data_smr_train <- subset(data_smr, NForGEN == 1)
data_smr_gen <- subset(data_smr, NForGEN == 2)
```

Linear mixed-effects-models (LMM)

Checking assumptions and defining final version Normality Diagnoses for both training and generalization sessions show a right-skewed fat tails distribution. Therefore, absolute EEG power values were log-transformed, and log-transformed values were used for statistical analysis.

```
plot_normality(data_smr_train, eeg_power)
```

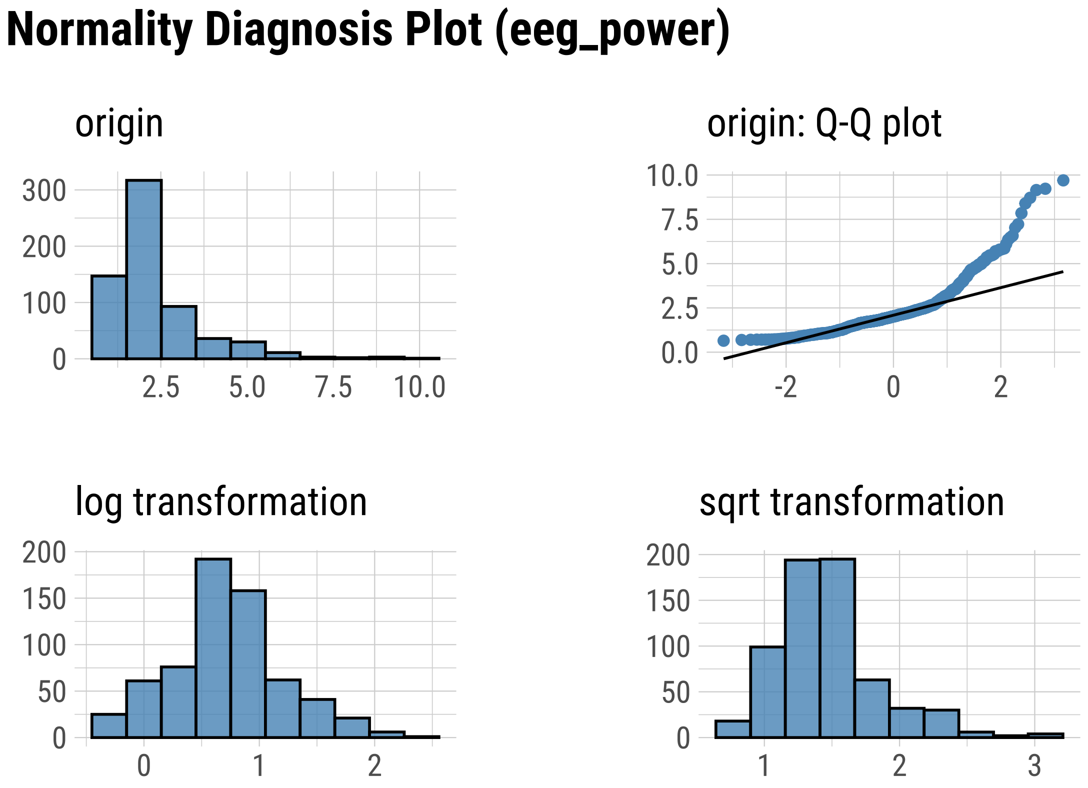


```
plot_normality(data_smr_gen, eeg_power)
```

Normality Diagnosis Plot (eeg_power)

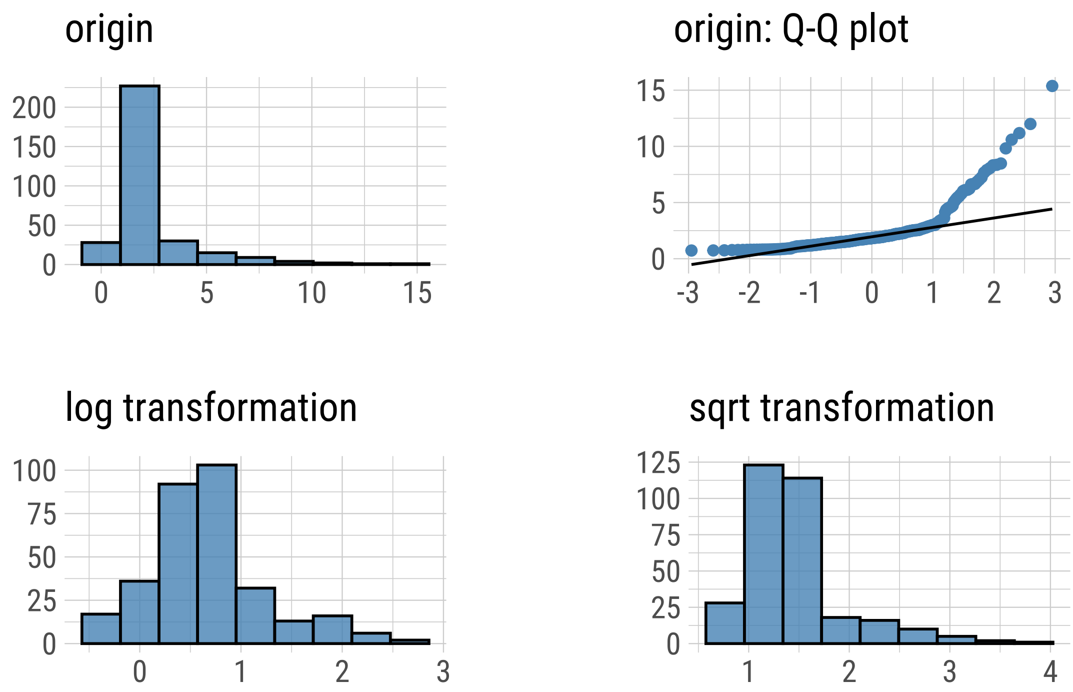


```
data_smr_train$log_power <- log(data_smr_train$eeg_power)
data_smr_gen$log_power <- log(data_smr_gen$eeg_power)
```

Now, the full LMM is calculated for training sessions: log-transformed SMR power as dependent variable, fixed effects: group, session (session 1-6), run (baseline, run 1-6) as triple-interaction, session and run as by-subject random slopes

```
train1 <- lmer(log_power ~ group * session_n * run_n + (session_n | subject) + (run_n | subject),
               data = data_smr_train, REML = FALSE)
```

```
## Warning in checkConv(attr(opt, "derivs"), opt$par, ctrl = control$checkConv, :
## unable to evaluate scaled gradient
```

```
## Warning in checkConv(attr(opt, "derivs"), opt$par, ctrl = control$checkConv, :
## Model failed to converge: degenerate Hessian with 1 negative eigenvalues
```

```
## Warning: Model failed to converge with 1 negative eigenvalue: -2.0e-04
```

This model failed to converge. It can be assumed, that there are not enough data points to calculate the full model. Therefore, the numerical variables *session* and *run* were converted into dichotomous variables.

```
data_smr_train$run_early_late <- NA
data_smr_train$run_early_late[data_smr_train$run_n %in% c(1:4)] <- 1 # early runs
data_smr_train$run_early_late[data_smr_train$run_n %in% c(5:7)] <- 2 # late runs
data_smr_train$run_early_late <- as.factor(data_smr_train$run_early_late)
```

```r
data_smr_train$ses_early_late <- NA
data_smr_train$ses_early_late[data_smr_train$session_n %in% c(2:4)] <- 1 # early sessions
data_smr_train$ses_early_late[data_smr_train$session_n %in% c(5:7)] <- 2 # late sessions
data_smr_train$ses_early_late <- as.factor(data_smr_train$ses_early_late)
```

Now, a reduced LMM with the new dichotomous variables is calculated:

```r
train2 <- lmer(log_power ~ group * ses_early_late * run_early_late +
               (ses_early_late | subject) + (run_early_late | subject),
               data = data_smr_train, REML = FALSE)
```

```
## boundary (singular) fit: see help('isSingular')
```

```r
isSingular(train2)
```

```
## [1] TRUE
```

Convergence was successful but unfortunately, there was a problem with singularity. Let's check the summary of the model:

```r
print(VarCorr(train2), comp = c("Variance", "Std.Dev."))
```

```
##  Groups    Name            Variance   Std.Dev. Corr
##  subject   (Intercept)     0.03476718 0.186460
##            ses_early_late2 0.02623456 0.161971 -0.467
##  subject.1 (Intercept)     0.13532455 0.367865
##            run_early_late2 0.00076847 0.027721 -1.000
##  Residual                  0.04752398 0.218000
```

You can see a perfect negative correlation of r = -1 for *subject + run_early_late*. It can be assumed that with the existing data points parameters of the model could not be estimated properly. At the moment, there is no consensus about how to deal with singularity, but according to Matuschek et al. (2017), singularity should be avoided, and LMM should be restricted to avoid fitting overly complex models. Therefore, we excluded the by-subject random slope *run_early_late*.

LMM: Training outcome This is the final LMM for training sessions:

```r
train_final <- lmer(log_power ~ group * ses_early_late * run_early_late +
                    (ses_early_late | subject), data = data_smr_train, REML = FALSE)
summary(train_final)
```

```
## Linear mixed model fit by maximum likelihood . t-tests use Satterthwaite's
##   method [lmerModLmerTest]
## Formula:
## log_power ~ group * ses_early_late * run_early_late + (ses_early_late |
##     subject)
##    Data: data_smr_train
##
##      AIC      BIC   logLik deviance df.resid
```

```
##      4.7     58.3      9.6    -19.3      631
##
## Scaled residuals:
##     Min      1Q  Median      3Q     Max
## -3.6618 -0.5600 -0.0763  0.4990  3.9266
##
## Random effects:
##  Groups   Name            Variance Std.Dev. Corr
##  subject  (Intercept)     0.15129  0.3890
##           ses_early_late2 0.02622  0.1619   -0.07
##  Residual                 0.04773  0.2185
## Number of obs: 643, groups:  subject, 17
##
## Fixed effects:
##                                           Estimate Std. Error        df t value
## (Intercept)                                0.58383    0.13156  17.43776   4.438
## groupExp                                   0.24403    0.19181  17.44716   1.272
## ses_early_late2                            0.02005    0.06238  21.08483   0.321
## run_early_late2                            0.05115    0.03445 609.28701   1.485
## groupExp:ses_early_late2                   0.02407    0.09116  21.27800   0.264
## groupExp:run_early_late2                  -0.05748    0.05000 609.12225  -1.150
## ses_early_late2:run_early_late2           -0.07505    0.04773 609.22538  -1.573
## groupExp:ses_early_late2:run_early_late2   0.08013    0.06995 609.21938   1.146
##                                          Pr(>|t|)
## (Intercept)                              0.000341 ***
## groupExp                                 0.219976
## ses_early_late2                          0.751066
## run_early_late2                          0.138144
## groupExp:ses_early_late2                 0.794323
## groupExp:run_early_late2                 0.250765
## ses_early_late2:run_early_late2          0.116344
## groupExp:ses_early_late2:run_early_late2 0.252424
## ---
## Signif. codes:  0 '***' 0.001 '**' 0.01 '*' 0.05 '.' 0.1 ' ' 1
##
## Correlation of Fixed Effects:
##              (Intr) grpExp ss_r_2 rn_r_2 grpExp:s__2 grpExp:r__2 s__2:_
## groupExp     -0.686
## ses_rly_lt2 -0.121  0.083
## run_rly_lt2 -0.109  0.075  0.229
## grpExp:s__2  0.083 -0.121 -0.684 -0.157
## grpExp:r__2  0.075 -0.109 -0.158 -0.689  0.230
## ss_rl_2:__2  0.078 -0.054 -0.326 -0.722  0.223       0.497
## grE:__2:__2 -0.054  0.078  0.223  0.493 -0.328      -0.715      -0.682
```

```
anova(train_final, type = "I")
```

```
## Type I Analysis of Variance Table with Satterthwaite's method
##                                  Sum Sq  Mean Sq NumDF  DenDF F value
## group                          0.081154 0.081154     1  17.02  1.7003
## ses_early_late                 0.006026 0.006026     1  16.94  0.1263
## run_early_late                 0.003209 0.003209     1 609.48  0.0672
## group:ses_early_late           0.021642 0.021642     1  16.96  0.4534
## group:run_early_late           0.009797 0.009797     1 609.21  0.2052
```

```
## ses_early_late:run_early_late       0.055837 0.055837     1 609.22  1.1699
## group:ses_early_late:run_early_late 0.062638 0.062638     1 609.22  1.3123
##                                     Pr(>F)
## group                               0.2096
## ses_early_late                      0.7267
## run_early_late                      0.7955
## group:ses_early_late                0.5098
## group:run_early_late                0.6507
## ses_early_late:run_early_late       0.2799
## group:ses_early_late:run_early_late 0.2524
```

LMM: Generalization To calculate comparable models the same LMM as above was used for the generalization sessions.

```
# converting session to dichotomous variable
data_smr_gen$ses_early_late <- NA
data_smr_gen$ses_early_late[data_smr_gen$session_n == 1] <- 1 # early session
data_smr_gen$ses_early_late[data_smr_gen$session_n %in% c(8:9)] <- 2 # late sessions
data_smr_gen$ses_early_late <- as.factor(data_smr_gen$ses_early_late)

# converting run to dichotomous variable
data_smr_gen$run_early_late <- NA
data_smr_gen$run_early_late[data_smr_gen$run_n %in% c(1:4)] <- 1 # early runs
data_smr_gen$run_early_late[data_smr_gen$run_n %in% c(5:7)] <- 2 # late runs
data_smr_gen$run_early_late <- as.factor(data_smr_gen$run_early_late)
```

```
gen_final <- lmer(log_power ~ group * ses_early_late * run_early_late +
                  (ses_early_late | subject), data = data_smr_gen, REML = FALSE)
summary(gen_final)
```

```
## Linear mixed model fit by maximum likelihood . t-tests use Satterthwaite's
##   method [lmerModLmerTest]
## Formula:
## log_power ~ group * ses_early_late * run_early_late + (ses_early_late |
##     subject)
##    Data: data_smr_gen
##
##      AIC      BIC   logLik deviance df.resid
##    187.8    232.9    -81.9    163.8      305
##
## Scaled residuals:
##     Min      1Q  Median      3Q     Max
## -2.8424 -0.4777 -0.0293  0.3573  5.1998
##
## Random effects:
##  Groups   Name            Variance Std.Dev. Corr
##  subject  (Intercept)     0.21088  0.4592
##           ses_early_late2 0.07575  0.2752   -0.49
##  Residual                 0.07399  0.2720
## Number of obs: 317, groups:  subject, 17
##
## Fixed effects:
```

```
##                                                Estimate Std. Error         df
## (Intercept)                                    0.571676   0.160851  18.494335
## groupExp                                       0.205952   0.237253  18.877593
## ses_early_late2                               -0.108715   0.109514  21.458953
## run_early_late2                               -0.047068   0.074089 284.580593
## groupExp:ses_early_late2                       0.382309   0.164176  20.891675
## groupExp:run_early_late2                      -0.014459   0.107960 284.397148
## ses_early_late2:run_early_late2                0.001259   0.090085 284.447423
## groupExp:ses_early_late2:run_early_late2      -0.125298   0.131998 284.384838
##                                               t value Pr(>|t|)
## (Intercept)                                     3.554  0.00219 **
## groupExp                                        0.868  0.39626
## ses_early_late2                                -0.993  0.33192
## run_early_late2                                -0.635  0.52575
## groupExp:ses_early_late2                        2.329  0.03000 *
## groupExp:run_early_late2                       -0.134  0.89356
## ses_early_late2:run_early_late2                 0.014  0.98886
## groupExp:ses_early_late2:run_early_late2       -0.949  0.34330
## ---
## Signif. codes:  0 '***' 0.001 '**' 0.01 '*' 0.05 '.' 0.1 ' ' 1
##
## Correlation of Fixed Effects:
##             (Intr) grpExp ss_r_2 rn_r_2 grpExp:s__2 grpExp:r__2 s__2:_
## groupExp    -0.678
## ses_rly_lt2 -0.533  0.362
## run_rly_lt2 -0.202  0.137  0.296
## grpExp:s__2  0.356 -0.547 -0.667 -0.198
## grpExp:r__2  0.138 -0.197 -0.203 -0.686  0.285
## ss_rl_2:__2  0.166 -0.113 -0.359 -0.823  0.239       0.565
## grE:__2:__2 -0.113  0.161  0.245  0.561 -0.346      -0.818      -0.682
```

```
anova_stats(gen_final)
```

	sumsq	meansq	NumDF	DenDF	statistic	p.value	partial.etasq
group	0.334	0.334	1	17.211	4.517	0.048	0.015
ses_early_late	0.026	0.026	1	16.218	0.355	0.560	0.001
run_early_late	0.641	0.641	1	284.648	8.665	0.004	0.029
group:ses_early_late	0.338	0.338	1	16.204	4.574	0.048	0.016
group:run_early_late	0.183	0.183	1	284.614	2.472	0.117	0.009
ses_early_late:run_early_late	0.055	0.055	1	284.393	0.749	0.388	0.003
group:ses_early_late:run_early_late	0.067	0.067	1	284.385	0.901	0.343	0.003

```
## [1] "Residuals Sume of Squares:  21.275"
```

Next step: Performing post-tests to check the sign. interaction *group + ses_early_late*. Sign. interaction could not be confirmed by our post-tests.

```
emms <- emmeans(gen_final, ~ group + ses_early_late, lmer.df = "satterthwaite", adjust = "bonferroni")
```

```
## NOTE: Results may be misleading due to involvement in interactions
```

```r
# defining relevant contrasts, EG = experimental group, CG = control group
contrast(emms, list(
  'EG (before training) - EG (after training)' = c(0, 1, 0, -1),
  'CG (before training) - CG (after training)' = c(1, 0, -1, 0),
  'EG (before training) - CG (before training)' = c(1, -1, 0, 0),
  'EG (after training) - CG (after training)' = c(0, 0, 1, -1)
), adjust = "bonferroni")
```

```
##  contrast                                    estimate    SE   df t.ratio
##  EG (before training) - EG (after training)    -0.212 0.115 16.2  -1.833
##  CG (before training) - CG (after training)     0.108 0.102 16.4   1.056
##  EG (before training) - CG (before training)   -0.199 0.233 17.5  -0.854
##  EG (after training) - CG (after training)     -0.518 0.199 17.2  -2.603
##  p.value
##   0.3409
##   1.0000
##   1.0000
##   0.0738
##
## Results are averaged over the levels of: run_early_late
## Degrees-of-freedom method: satterthwaite
## P value adjustment: bonferroni method for 4 tests
```

Frequency analysis

In the end, we created for the frequency analysis two dataframes (experimental group and control group) including the total frequencies (column “frequency”) and frequency of users (column “docfreq”), separated for the data points (S01 - pre-training, S02 - first training, S07 - last training, S08 - 24 h post-training, S09 - 7 days post-training).

```r
strategies <- read_excel("data_n25_strategies.xlsx")

corp_all <- corpus(strategies, text_field = "strategy")
```

```
## Warning: NA is replaced by empty string
```

```r
docid <- paste(strategies$subject, strategies$session, sep = " ")
docnames(corp_all) <- docid

corp_EG <- corpus_subset(corp_all, group == "EG")
corp_CG <- corpus_subset(corp_all, group == "CG")

toks_CG <- corp_CG %>%
  tokens(remove_punct = TRUE) %>%
  tokens_remove(c(stopwords("de"), c("dass", "etc", "bzw"))) %>%
  tokens_keep(pattern = "^[\\p{script=Latn}]+$", valuetype = "regex")

toks_EG <- corp_EG %>%
  tokens(remove_punct = TRUE) %>%
  tokens_remove(c(stopwords("de"), c("dass", "etc", "bzw"))) %>%
  tokens_keep(pattern = "^[\\p{script=Latn}]+$", valuetype = "regex")
```

```
dfmat_CG <- dfm(toks_CG)
dfmat_EG <- dfm(toks_EG)

tstat_freq_CG <- dfmat_CG %>%
  textstat_frequency(groups = session) %>%
  as.data.frame()

tstat_freq_EG <- dfmat_EG %>%
  textstat_frequency(groups = session) %>%
  as.data.frame()

head(tstat_freq_CG)
```

```
##          feature frequency rank docfreq group
## 1          kreuz         5    1       3   S01
## 2       versucht         3    2       3   S01
## 3         zählen         3    2       3   S01
## 4         denken         3    2       3   S01
## 5          punkt         3    2       2   S01
## 6 konzentrieren         3    2       3   S01
```

```
head(tstat_freq_EG)
```

```
##    feature frequency rank docfreq group
## 1  möglich         6    1       4   S01
## 2 versucht         6    1       5   S01
## 3    kreuz         6    1       5   S01
## 4    runde         3    4       1   S01
## 5     zeit         3    4       2   S01
## 6    immer         3    4       2   S01
```

Literature

Matuschek, H., Kliegl, R., Vasishth, S., Baayen, H. & Bates, D. (2017). Balancing Type I error and power in linear mixed models. *Journal of Memory and Language, 94, 305– 315.* https://doi.org/10.1016/j.jml.2017.01.001
