# Supplementary material for: Assessment of the capacity to modulate brain signals in a home-based SMR neurofeedback training setting
Source: Front Hum Neurosci. 2023 Jan 5;16:1032222. doi: 10.3389/fnhum.2022.1032222 (PMC9849904; doi:10.3389/fnhum.2022.1032222)
Supplement: Supplementary file 3 [file Data_Sheet_3.pdf]

Supplementary Material C

The results for the mixed-effects models for the dependent variables Beta power and Theta power are presented in Table 1 and Table 2, respectively.

Beta power (Table 1, Figure 1): No significant changes in Beta power within and across NFT sessions were shown by any of the two groups. There was a significant main effect run in the generalization sessions for the dependent variable Beta power (η^2_p = 0.016). Additionally, there was a significant interaction session * run (η^2_p = 0.017). This is in line with Kober et al. (2015) and shows that Beta power is not independent of SMR power modulation.

Theta power (Table 2, Figure 2): No significant changes were found in Theta power, neither in NFT sessions nor in generalization sessions.

Table 1

Results of the mixed-effects models with the linear effects group (experimental or control group), session (early vs. late sessions), and run (early vs. late runs), and the by-subject random slope session (early vs. late sessions) for the dependent variable log-transformed Beta power, presented separately for the training and generalization sessions.

		**NFT sessions**			**Generalization sessions**		
		F (df, dfError)	***MSE***	***p-value (η^2_p)***	***F (df, dfError)***	***MSE***	***p-value (η^2_p)***
log-Beta Power	Group	0.02 (1, 16.98)	0.002	0.90	1.02 (1, 17.15)	0.10	0.33
	Session	0.41 (1, 17.35)	0.04	0.53	3.08 (1, 15.76)	0.29	0.10
	Run	2.70 (1, 609.98)	0.26	0.10	4.61 (1, 284.51)	0.43	0.03 * (0.016)
	Group * Session	1.51 (1, 17.37)	0.15	0.24	0.67 (1, 15.73)	0.06	0.42
	Group * Run	0.38 (1, 609.79)	0.04	0.54	0.77 (1, 284.47)	0.07	0.38
	Session * Run	0.11 (1, 609.80)	0.01	0.74	5.00 (1, 284.12)	0.46	0.03 * (0.017)
	Group * Session * Run	1.12 (1, 609.80)	0.11	0.29	0.26 (1, 284.11)	0.02	0.61

Note. Significant results are marked with * (* $p < 0.05$).

Table 2

Results of the mixed-effects models with the linear effects group (experimental or control group), session (early vs. late sessions), and run (early vs. late runs), and the by-subject random slope session (early vs. late sessions) for the dependent variable log-transformed Theta power, presented separately for the training and generalization sessions.

		**NFT sessions**			**Generalization sessions**		
		F (df, dfError)	***MSE***	***p-value***	***F (df, dfError)***	***MSE***	***p-value***
log-Theta Power	Group	0.01 (1, 17.00)	0.0004	0.92	1.41 (1, 17.06)	0.11	0.25
	Session	1.76 (1, 17.19)	0.07	0.20	1.54 (1, 16.04)	0.12	0.23
	Run	0.0009 (1, 609.69)	0.00003	0.98	0.64 (1, 283.41)	0.05	0.43
	Group * Session	0.33 (1, 17.21)	0.01	0.57	2.86 (1, 16.09)	0.23	0.11
	Group * Run	1.67 (1, 609.44)	0.06	0.20	1.89 (1, 283.36)	0.15	0.17
	Session * Run	1.19 (1, 609.44)	0.05	0.28	0.93 (1, 283.18)	0.08	0.34
	Group * Session * Run	0.41 (1, 609.44)	0.02	0.52	2.63 (1, 283.16)	0.21	0.11

Figure 1

Within- and between-session changes in log-transformed Beta power for training and generalization sessions, separately presented for experimental (blue) and control group (orange).

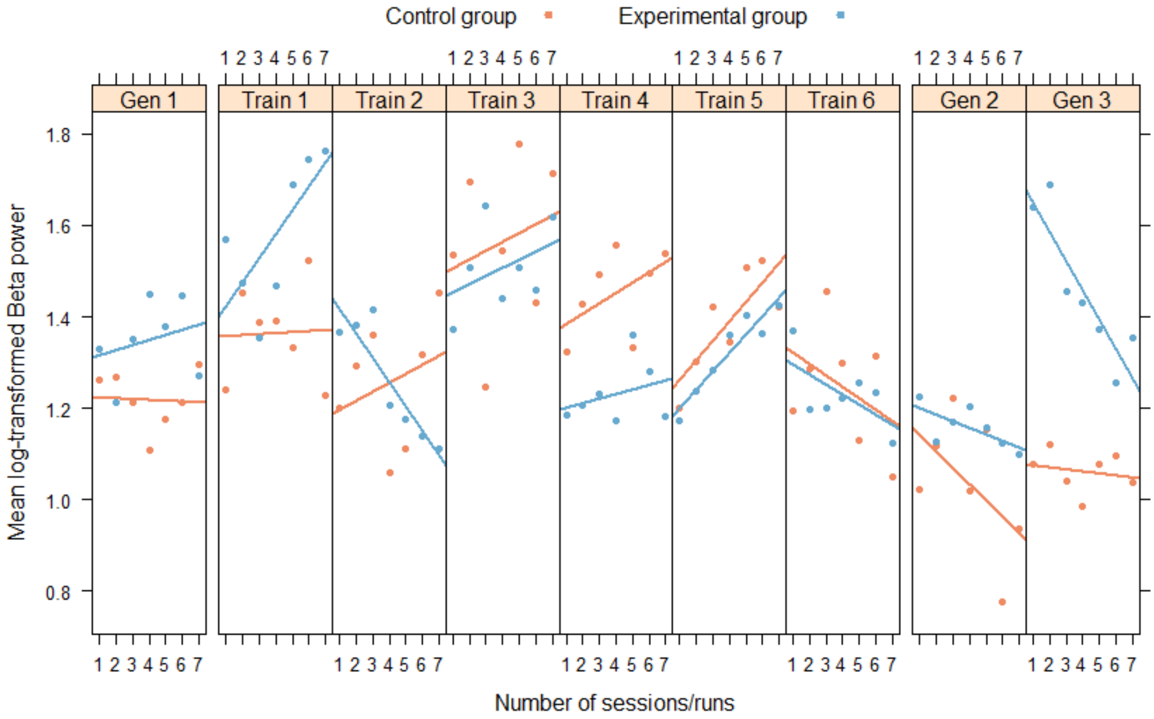

Figure 2

Within- and between-session changes in log-transformed Theta power for training and generalization sessions, separately presented for experimental (blue) and control group (orange).

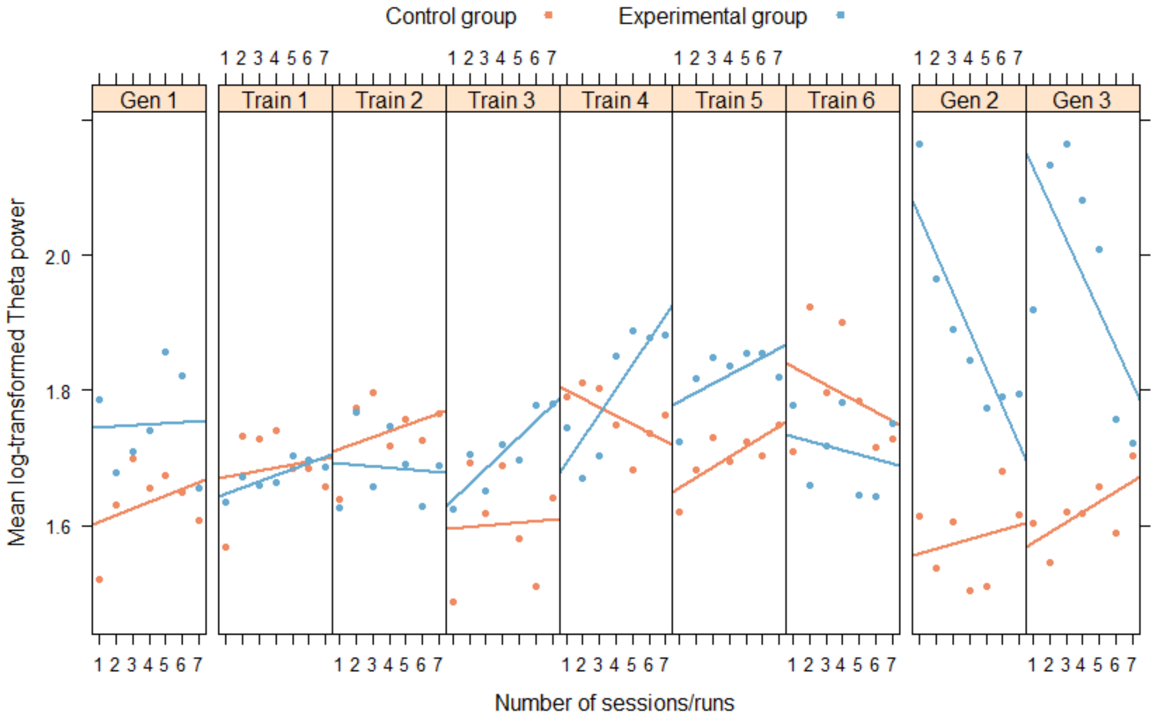

Literature

Kober, S. E., Witte, M., Stangl, M., Väljamäe, A., Neuper, C., & Wood, G. (2015). Shutting down sensorimotor interference unblocks the networks for stimulus processing: An SMR neurofeedback training study. *Clinical Neurophysiology*, *126*(1), 82-95.
